# Investigation on Fabrication of Reduced Graphene Oxide-Sulfur Composite Cathodes for Li-S Battery via Hydrothermal and Thermal Reduction Methods

**DOI:** 10.3390/ma14040861

**Published:** 2021-02-11

**Authors:** Zhiqi Li, Hao Sun, Yuepeng Pang, Mingming Yu, Shiyou Zheng

**Affiliations:** 1 School of Materials Science and Engineering, University of Shanghai for Science and Technology, Shanghai 200093, China; lizhiqiusst@163.com (Z.L.); sunhao@usst.edu.cn (H.S.); pangyp@usst.edu.cn (Y.P.); 2 Research Center of Composite Materials, School of Materials Science and Engineering, Shanghai University, Shanghai 200000, China

**Keywords:** reduced graphene oxide, sulfur, composite, battery, Li-S battery

## Abstract

Lithium-sulfur (Li-S) battery is considered one of the possible alternatives for next-generation high energy batteries. However, its practical applications are still facing great challenges because of poor electronic conductivity, large volume change, and polysulfides dissolution inducing “shuttle reaction” for the S cathode. Many strategies have been explored to alleviate the aforementioned concerns. The most common approach is to embed S into carbonaceous matrix for constructing C-S composite cathodes. Herein, we fabricate the C-S cathode reduced graphene oxide-S (rGO-S) composites via one step hydrothermal and in-situ thermal reduction methods. The structural features and electrochemical properties in Li-S cells of the two type rGO-S composites are studied systematically. The rGO-S composites prepared by one step hydrothermal method (rGO-S-HT) show relatively better comprehensive performance as compared with the ones by in-situ thermal reduction method (rGO-S-T). For instance, with a current density of 100 mA g^−1^, the rGO-S-HT composite cathodes possess an initial capacity of 1290 mAh g^−1^ and simultaneously exhibit stable cycling capability. In particular, as increasing the current density to 1.0 A g^−1^, the rGO-S-HT cathode retains a reversible capacity of 582 mAh g^−1^ even after 200 cycles. The enhanced electrochemical properties can be attributed to small S particles uniformly distributed on rGO sheets enabling to significantly improve the conductivity of S and effectively buffer large volume change during lithiation/delithiation.

## 1. Introduction

Lithium-sulfur (Li-S) batteries have a hopeful prospect among emerging battery systems because of their high specific capacity and energy density in theory [1,2,3,4,5]. Unfortunately, their practical realization suffers from low active material utilization and poor cycle life together with low Coulombic efficiency, which are mainly related to electrically insulating characteristics of S and its discharging products (Li_2_S_2_ and Li_2_S), solubility of the reaction intermediates polysulfides (Li_2_S*x*, 3 ≤ *x* ≤ 8) in liquid electrolytes, shuttle reaction of dissolved lithium polysulfides, and large volume variation during lithiation/delithiation [6,7,8,9,10].

To alleviate the aforementioned problems, many research attempts have been conducted to design special structured materials in order to construct S-based cathodes, functional interlayers, hybrid electrolytes, and modified separators for Li-S batteries. The electrolytes with different additives have been recognized to enhance the stability of electrochemical reactions during charge and discharge process [11,12,13]. Some functional materials are adopted as interlayer or coated to separator, which could suppress shuttling reaction effectively [14,15,16]. Most of the research is dedicated to designing and constructing S-based cathode materials, focusing on loading the active material S in a variety of matrix, such as carbon-based materials, conductive polymers, transition metal oxide, and 2D Ti_3_C_2_T_x_ (MXenes), etc. [17,18,19,20,21,22,23,24,25]. The most common strategy is to embed S into the carbonous matrix. Various carbon materials with different microstructures have been investigated to enhance the conductivity and simultaneously to prevent polysulfide intermediates from dissolving into liquid electrolytes [26,27,28,29,30]. Over the past few years, graphene has been considered an ideal host for loading active material S to fabricate high-performance composite cathodes owing to unique features of graphene such as high surface area and electrical conductivity [31,32,33,34,35,36,37,38,39]. The graphene is usually prepared based on Hummers’ oxidation and reduction method [40]. The preparation of composite cathodes involves the reduction of graphene oxide (rGO) into graphene together with S loading. For instance, many researchers reported that oxygen-containing functional groups on the GO can immobilize S species by chemisorption and thus improve cycling performance of the S-based cathodes [41,42]. Zhang and co-workers synthesized a layered structural composite of S/ polypyrrole/graphene, presenting high active material S usage and cycling stability [43]. Furthermore, the S-rGO composite was synthesized through bubbling hydrogen sulfide gas into GO suspension, which can implement the GO reduction and form the graphene-S composite simultaneously [44]. Although the GO is the most commonly employed as precursor for preparing rGO and its derivatives, it usually possesses low conductivity because of the existence of a lot of oxygen-containing functional groups leading to the original sp2 hybridized network destruction. In particular, the graphene-S cathodes fabricated through different methods will not present equally electrochemical performance in Li-S battery. It is worth studying the correlation between the property of the cathodes and the fabrication approach.

Herein, we fabricate the reduced graphene oxide-S (rGO-S) composite cathodes via two common methods, i.e., one step hydrothermal and in-situ thermal reduction. The structural features and electrochemical properties in Li-S cells of the two types of as-prepared rGO-S composites are studied systematically. Our investigation demonstrates that the rGO-S composites prepared by one step hydrothermal method (rGO-S-HT) have relatively better comprehensive performance as compared with the ones by in-situ thermal reduction method (rGO-S-T).

## 2. Materials and Methods

### 2.1. Synthesis of Graphene Oxide (GO) 

GO was synthesized by oxidation of natural graphite powders (Sigma-Aldrich, Shanghai, China, particle size <20 μm) referring to the modified Hummers’ approaches [40]. The as-prepared GO was firstly dispersed in purified H_2_O and then exfoliated by 30 min ultrasonication treatment, following with a centrifugation process at 5000 r.p.m for 30 min. Finally, the GO powders used in this work were obtained through vacuum freeze-drying technique.

### 2.2. Synthesis of rGO-S Composites by One Step Hydrothermal Method (rGO-S-HT)

The rGO-S-HT composites were prepared by one step hydrothermal method, i.e., the as-synthesized GO colloidal suspension (5 mL, 5 mg mL^−1^), Na_2_S_2_O_3_ (5 M, 5 mL), and HCl (1M, 3 mL) were put into a Teflon-lined autoclave to stir for 30 min, and then subjected to react at the ambient temperature of 180 °C for 12 h in vacuum oven to ensure chemical self-assembly reaction finished. The rGO-S-HT powder sample was obtained by multiple cleaning-filtration and final vacuum freeze drying. All chemical reagents used in this work were purchased from Sigma-Aldrich (Shanghai, China).

### 2.3. Synthesis of rGO-S Composites by In-Situ Thermal Reduction Method (rGO-S-T)

The rGO-S-T composites were prepared by in-situ thermal reduction method. Firstly, the as-synthesized GO powder and the pristine sublimed sulfur (Sigma-Aldrich, Shanghai, China) were weighed with a mass ratio of 2:1 and added in an agate mortar for ground milling. Then, the mixture was reactive sintered in a muffle furnace by sealing in an evacuated quartz tube with the following conditions: (i) heating up from room temperature to high temperature of 600 °C, (ii) holding at this temperature for 6 h, and (iii) cooling down from high temperature to room temperature at a slow rate of 0.5 °C min^−1^. Finally, the rGO-S-T powder sample was obtained by opening the quartz tube.

### 2.4. Characterizations

The sulfur content in the composite was obtained using thermogravimetric analyzer (TGA) on a Netzsch STA 449 F1 (Selb, Germany) with the measurement conditions: (i) temperature range: 50–600 °C; (ii) heating rate: 10 °C min^−1^; and (iii) purge gas: high purity N_2_. The X-ray diffraction (XRD) patterns of the rGO-S and the pristine S samples were recorded in the 2-theta range from 10–80° using Rigaku D/max 2400 (Rigaku, Tokyo, Japan) with Cu Kα radiation. The Raman spectra of samples were obtained on a dispersive Raman spectroscopic microscope (Horiba Jobin Yvon, Atlanta, GA, USA). The X-ray photoelectron spectroscopy (XPS) was carried out on an upgraded PHI-5000C ESCA system (Perkin Elmer, Hopkinton, MA, USA) with Al Kα X-ray spectrometer at 14 kV. Scanning electron microscope (SEM) was used to observe morphology of samples on Hitachi S-4700 (Hitachi, Tokyo, Japan) operating at 10 kV, meanwhile equipped with an energy dispersive X-ray spectrometry (EDS) (Oxford, Abingdon, UK) to characterize the elemental mapping.

### 2.5. Electrode Preparation and Electrochemical Measurements

Cathode slurry was firstly prepared by weighing the active material rGO-S composites, conductive carbon black (super P), and binder poly(vinylidene fluoride) (PVDF) with a mass ratio of 8:1:1 and adding in an agate mortar to grind for 30 min. Then, certain volume solvent N-methy-1-2-pyrrolidone (NMP) was added to the mixture following with magnetically stirring overnight. Finally, the electrode film was coated by casting the obtained slurry onto an aluminum foil and vacuum-oven drying at 60 °C no less than 12 h. The rGO-S films were cut into round pieces with a diameter of 1.2 cm by precision disc cutter machine, which were used as the Li-S cell electrode directly. Our fabricated electrode film contained the active material S of around 2 mg cm^−2^. Lithium metal was employed as the counter electrode and reference electrode, and the microporous polypropylene Celgard 3501 (Celgard, LLC Corp., Charlotte, NC, USA) as separator adopted 1.0 M bis-(trifl uoromethane) sulfonimide lithium salt (LiTFSI, Sigma-Aldrich, Shanghai, China) dissolved in a mixture of 1,3-dioxolane (DOL) and dimethoxyethane (DME) (DOL/DME, 1:1 v/v) (Sigma-Aldrich, Shanghai, China) was adopted as Li-S battery electrolyte. The coin cells were assembled in a glove box filled with high pure Ar gas. The galvanostatic charge-discharge (LAND CT 2001, Wuhan, China) and cyclic voltammetry tests (CHI660c Electrochemical Workstation, Shanghai, China) were carried out to detect electrochemical properties. The specific capacity was calculated on the basis of the active material S tested by TGA measurement.

## 3. Results and Discussion

### 3.1. Morphology

**Figure 1:**
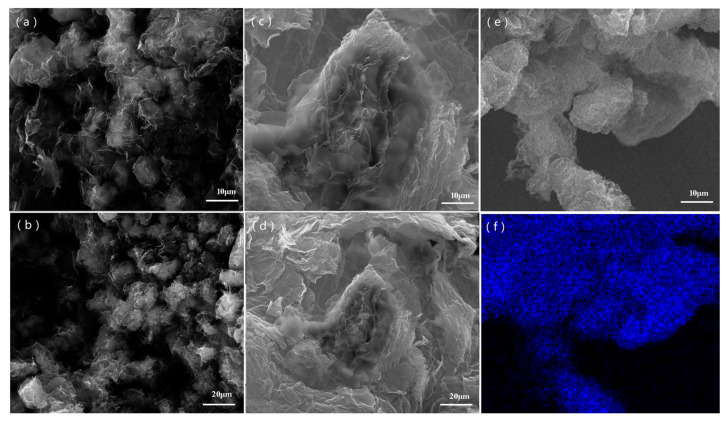
SEM images of rGO-S-HT (**a**,**b**), rGO-S-T (**c**,**d**), and SEM image with corresponding elemental mapping of rGO-S-HT for S (**e**,**f**).

SEM were conducted to observe morphology of the rGO-S composite materials. The SEM images of the rGO-S-HT and rGO-S-T are depicted in Figure 1. As shown in Figure 1a,b, it can be seen that small S particles were distributed uniformly on graphene layer for the rGO-S-HT sample. While for the rGO-S-T sample (see Figure 1c,d), the sheet feature structural rGO is also observed, but the S particles have more obvious aggregation. It is well known that the rGO sheets can serve as immobilizer to anchor S and to protect polysulfide intermediates from dissolution into electrolytes [30,31,32,33,34,35]. Meanwhile, the S particles’ distribution in graphene layers will facilitate improvement of the conductivity of the S. Moreover, the graphene possesses a robust structure with good flexibility and strength, which enables tolerating severe volume change for the S during lithiation/delithiation. The EDS elemental mapping for the rGO-S-HT is also displayed in Figure 1e and f. The S map follows the structure of the rGO, which suggests that the S particles are well-dispersed in the rGO-S-HT composite.

### 3.2. Structural Characteristics

**Figure 2:**
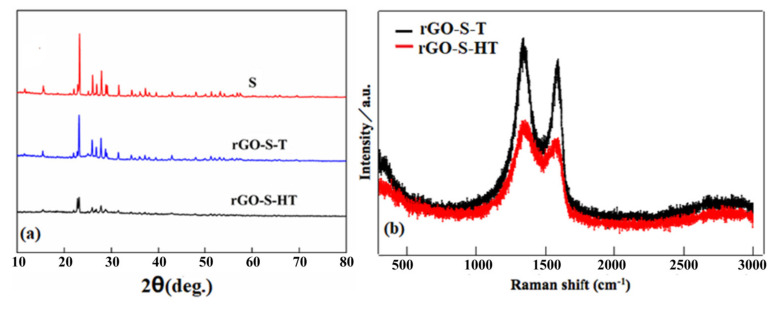
(**a**) XRD patterns of S, reduced graphene oxide-sulfur by in-situ thermal reduction (rGO-S-T) and rGO-S-HT (by hydrothermal method) samples, (**b**) Raman spectra of rGO-S-T and rGO-S-HT samples.

Figure 2a shows the XRD patterns of S, rGO-S-HT and rGO-S-T samples. The pristine S powder sample presents sharp and strong peaks curve, suggesting its typical S_8_ crystal structure. For the rGO-S-T, all the diffraction peaks are almost matching with the S patterns, that is, the peaks from the crystalline S are obvious, which means that the S deposited on the rGO sheets is mainly in form of S_8_ because the small S molecules will re-associate and finally form cyclo-S_8_ as the temperature cools down during the rGO-S composite preparing process by in-situ thermal reduction [33]. In contrast, the rGO-S-HT shows much weaker diffraction peak intensity than the rGO-S-T sample, which indicates that the form of S is small molecular and/or amorphous. Raman spectroscopy is commonly used to characterize the graphene and other carbon materials. The Raman spectra of rGO-S-T and rGO-S-HT are shown in Figure 2b. There are two distinct peaks both for the rGO-S-T and rGO-S-HT, arising from well-known G and D bands. In addition to similar peak positions, the D/G band intensity ratios (I_D_/I_G_) exceed 1.0, indicating perfect degree of reduction from GO to rGO both for rGO-S-T and rGO-S-HT composite [30,31,38].

**Figure 3:**
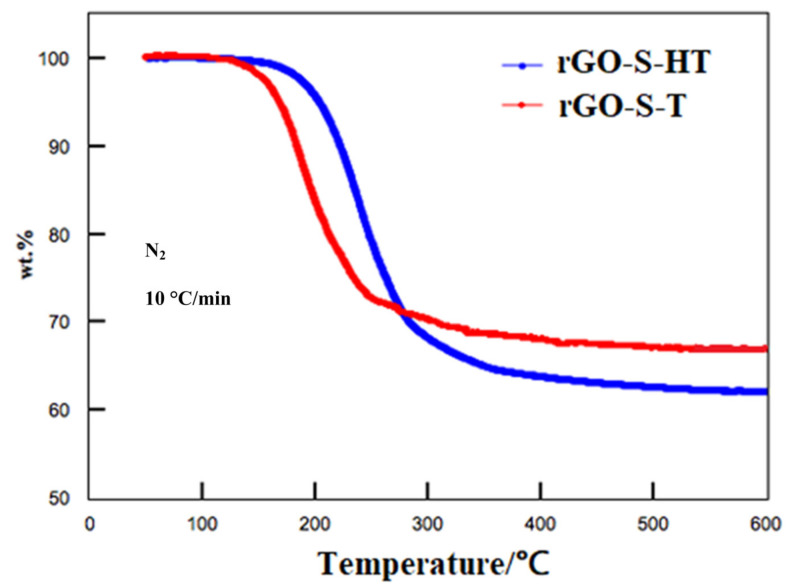
TGA curves of rGO-S-HT and rGO-S-T samples.

The S contents in the rGO-S composites were tested by TGA. Figure 3 displays the TGA profile of the two composites. Around 40% S was loaded in the rGO-S-HT composite. The value is higher than that of 32% for the rGO-S-T composite. This difference demonstrates that one step hydrothermal method is more beneficial for S loading. In addition, it is worth noting that the onset weight loss takes place at around 120 °C for the rGO-S-T composite with the entire weight loss at approximately 300 °C. This phenomenon is almost identical to the evaporation of S_8_. While for the rGO-S-HT composite, the TGA curve has two-step weight loss characteristic. At around 180 °C, the first weight loss starts and ~30% loss occurs as the temperature reaches to 300 °C. The second weight loss takes place with the progressive increasing temperature from 300 to around 500 °C, meanwhile, the rest of the loaded S is released. This step is considered in previously reported literatures as the extraction of small molecular and/or amorphous S [28,39].

**Figure 4:**
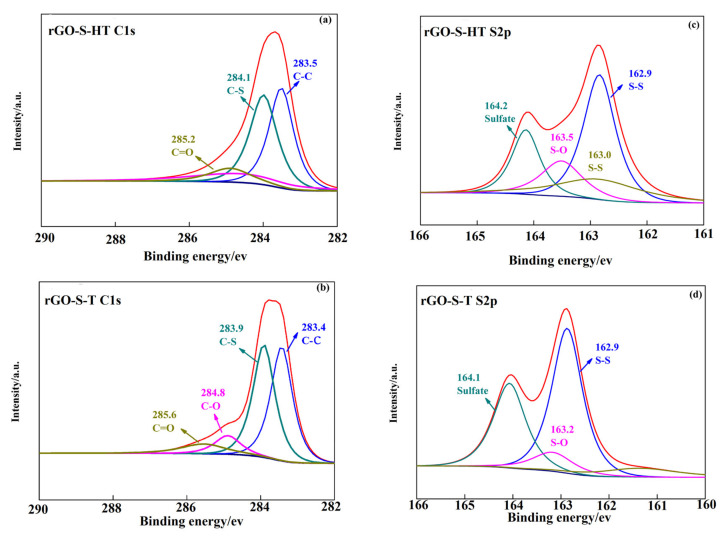
(**a**,**b**) C1’s signal obtained from XPS spectra of rGO-S-HT and rGO-S-T, (**c**,**d**) S 2p XPS spectra of rGO-S-HT and rGO-S-T.

The surface chemical compositions and functional groups of the rGO-S-HT and rGO-S-T were identified by XPS technique. Figure 4 presents the C1s and S 2p binding energy spectra of the two composite samples. The C1s exhibits one strong peak at about 284.0 eV for the rGO-S-HT. By further fitting, we can split the strong peak into three peaks 283.5, 284.1, and 285.2 eV, respectively, which are assigned to C–C, C–S, and C=O (Figure 4a). As for the rGO-S-T (Figure 4b), the C1s possesses one strong peak and a shou1der weak located at around 284.0 eV and at 285.0 eV, respectively, which suggests the existence of C-O bonding in rGO-S-T [45,46]. The S 2p has one strong peak of around 163.0 eV and a broad peak of 164.2 eV both for the two rGO-S composite samples (Figure 4c,d). While for the binding energy of S2p 3/2 peak, it is located at 162.9 eV for the two rGO-S composite samples, lower than the value of elemental S (164.0 eV), which reveals the existence of the chemical bonding between S and rGO. After further fitting, the peak at 163.5 eV can be attributed to the S–O bonds, and the peak at 164.2 eV may be ascribed to the sulphate species formed from sulfur oxidation between S and oxygen functional groups of GO [27,28,39,47].

### 3.3. Electrochemical Performance

**Figure 5:**
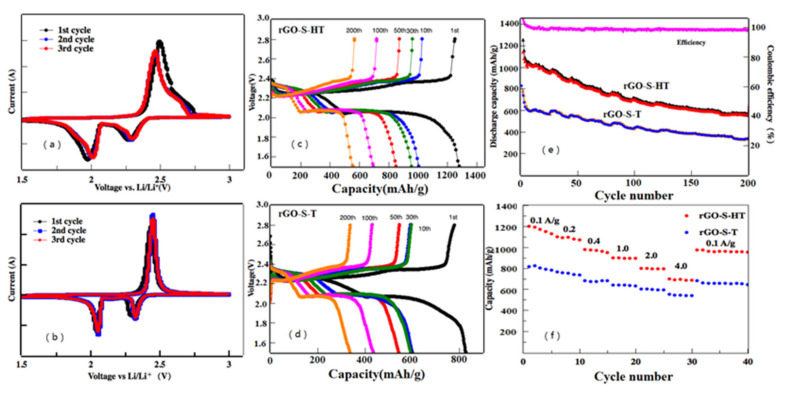
(**a**,**b**) Cyclic voltammograms (CVs) of the rGO-S-HT and rGO-S-T cathode at 0.1 mV s^−1^ with a potential window of 1.5–3 V vs Li^+^/Li, (**c**,**d**) Discharge/charge voltage profiles of the rGO-S-HT and rGO-S-T cathode for the 1st, 50th cycles at 0.1 A g^−1^, (**e**) Cyclic performance of the rGO-S-HT and rGO-S-T cathode at 1.0 A g^−1^, (**f**) Rate performance of the rGO-S-HT and rG-S-T cathode at different current densities from 0.1 to 4.0 A g^−1^.

The electrochemical performances of the as-obtained rGO-S-HT and rGO-S-T composite cathodes for Li-S battery were tested through cyclic voltammogram (CV) combining with galvanostatic charge/discharge measurement. Figure 5a,b shows the first three cycling CV curves of the rGO-S-HT and rGO-S-T cathodes at a sweep rate of 0.1 mV s^−1^ with the potential range of 1.5–3.0 V vs. Li^+^/Li. Two main peaks of around 2.3 and 2.0 V is clearly presented in the cathodic scan, ascribing to the transformation from cyclo-S_8_ to high-order lithium polysulfide intermediates (Li_2_S_n_, 4 ≤ n ≤ 8) and high-order lithium polysulfides into Li_2_S_2_ and Li_2_S, respectively. While in the anodic scan, only one strong oxidation peak located around 2.4 V is detected, which belongs to the coupled conversion from Li_2_S to lithium polysulfides Li_2_S_n_ (4 ≤ n ≤ 8) and ultimately to sulfur [8,9]. In addition, slight shifts are also observed from both the reduction and oxidation peaks in the following two cycles, which related to the polarization of the cathode materials [35]. The voltage profiles of the rGO-S-HT and rGO-S-T cathodes during the 1st, 10th, 30th, 50th, 100th, and 200th cycles at a current density of 0.1 A g^−1^ are shown in Figure 5c,d. From the first cyclic discharge curves, there are three voltage plateaus located at ~2.3, 2.1, and 1.8 V. They are attributed to the lithiation process of S_8_ to form high-order polysulfide intermediates (Li_2_S_n_, 4 ≤ n ≤ 8) and finally to produce insoluble low-order polysulfides Li_2_S_2_ and/or Li_2_S, which is in agreement with the observed peaks in the CV (see Figure 5c). The first lithiation capacity of the rGO-S-HT cathode is around 1290 mAh g^−1^. The rGO-S-T cathode in comparison in Figure 5d, however, has the similar voltage plateaus but releases lower discharge capacity. In particular, the rGO-S-HT cathodes present better cycling stability as compared with the rGO-S-T cathode with progressive cycling. For instance, the 100th cycling discharge capacity is less than 430 mAh g^−1^ while the rGO-S-HT cathode can still deliver the capacity of 580 mAh g^−1^ even after undergoing 200 cycles.

Figure 5e displays cyclic performance curve at 1.0 A g^−1^ for the rGO-S-HT and rGO-S-T cathodes. It can also deliver a reversible capacity of 582 mAh g^−1^ even after 200 cycles. Moreover, the Coulombic efficiency of rGO-S-HT is close to ~97%, demonstrating an effective limitation to the shuttling effect. By comparison, the rGO-S-T was also cycled under same electrochemical measurement conditions. It is obvious that the rGO-S-T cathode shows relatively worse cyclic lifespan than that of rGO-S-HT cathode. After 200 cycles, the capacity is less than 300 mAh g^−1^, which is almost half capacity of the rGO-S-HT. The rGO-S-HT cathode also exhibits good rate performance, as displayed in Figure 5f. The reversible specific capacities of the first cycle are 1197, 1100, 980, 901, 817, and 719 mAh g^−1^ at 0.1, 0.2, 0.4, 1, 2, and 4 A g^−1^ current densities, respectively, which is much higher than many of the S-based cathodes reported in the literature [17,19,23,24,25,26]. Furthermore, when the current density returns to 0.1 A g^−1^, the discharge capacity of rGO-S-HT cathode can return to 1012 mAh g^−1^, demonstrating good electrochemical reversibility for the rGO-S-HT cathode. As for the rGO-S-T cathode, it exhibits relatively poor rate performance.

Obviously, the rGO-S composites prepared by the one-step hydrothermal method show relatively better comprehensive performance as compared with the ones by in-situ thermal reduction method, and especially, the rGO-S-HT cathodes present good cycling stability and excellent rate performance. The enhanced electrochemical properties in Li-S battery can be attributed to small S particles uniformly distributed on the rGO sheets enabling to significantly improve the conductivity of S and effectively buffer large volume change during lithiation/delithiation. In addition, our results suggest that higher sulfur can be loaded in the rGO-S-HT, which is mainly related with the microstructure of the rGO due to different reduction methods. The internal mechanism will be further investigated in our future work.

## 4. Conclusions

In this work, two rGO-S composites were prepared via one step hydrothermal and in-situ thermal reduction methods. The structural features and electrochemical performance in Li-S cells of the composites are studied and compared. The rGO-S composites prepared by one-step hydrothermal method show relatively better comprehensive performance as compared with the ones by the in-situ thermal reduction method, demonstrating good cycling stability, and excellent rate performance in Li-S battery. The uniform distribution of small S particles on the rGO sheets is the key factor, which enables to significantly improve the conductivity of S and effectively buffer large volume change during lithiation/delithiation. Our results provide direct evidence that the electrochemical performance of the C-S composite electrodes for Li-S batteries are mainly related with the S uniformly confined by the unique structural matrix. The related mechanisms need to be further investigated in future work.

## Data Availability

The data presented in this study are available on request from the corresponding author.
